# Automatic Evaluation of Heart Condition According to the Sounds Emitted and Implementing Six Classification Methods

**DOI:** 10.3390/healthcare9030317

**Published:** 2021-03-12

**Authors:** Manuel A. Soto-Murillo, Jorge I. Galván-Tejada, Carlos E. Galván-Tejada, Jose M. Celaya-Padilla, Huizilopoztli Luna-García, Rafael Magallanes-Quintanar, Tania A. Gutiérrez-García, Hamurabi Gamboa-Rosales

**Affiliations:** 1 Unidad Académica de Ingeniería Eléctrica, Universidad Autónoma de Zacatecas, Jardín Juarez 147, Centro, Zacatecas 98000, Mexico; alejandro.somu@uaz.edu.mx (M.A.S.-M.); ericgalvan@uaz.edu.mx (C.E.G.-T.); jose.celaya@uaz.edu.mx (J.M.C.-P.); hlugar@uaz.edu.mx (H.L.-G.); tiquis@uaz.edu.mx (R.M.-Q.); hamurabigr@uaz.edu.mx (H.G.-R.); 2 Departamento de Ciencias Computacionales, Centro Universitario de Ciencias Exactas e Ingenierías, Universidad de Guadalajara, Blvd. Marcelino García Barragán 1421, Guadalajara, Jalisco 44430, Mexico; tania.gutierrez@academicos.udg.mx

**Keywords:** heart sounds, heart disease, classification methods, evaluation metrics

## Abstract

The main cause of death in Mexico and the world is heart disease, and it will continue to lead the death rate in the next decade according to data from the World Health Organization (WHO) and the National Institute of Statistics and Geography (INEGI). Therefore, the objective of this work is to implement, compare and evaluate machine learning algorithms that are capable of classifying normal and abnormal heart sounds. Three different sounds were analyzed in this study; normal heart sounds, heart murmur sounds and extra systolic sounds, which were labeled as healthy sounds (normal sounds) and unhealthy sounds (murmur and extra systolic sounds). From these sounds, fifty-two features were calculated to create a numerical dataset; thirty-six statistical features, eight Linear Predictive Coding (LPC) coefficients and eight Cepstral Frequency-Mel Coefficients (MFCC). From this dataset two more were created; one normalized and one standardized. These datasets were analyzed with six classifiers: k-Nearest Neighbors, Naive Bayes, Decision Trees, Logistic Regression, Support Vector Machine and Artificial Neural Networks, all of them were evaluated with six metrics: accuracy, specificity, sensitivity, ROC curve, precision and F1-score, respectively. The performances of all the models were statistically significant, but the models that performed best for this problem were logistic regression for the standardized data set, with a specificity of 0.7500 and a ROC curve of 0.8405, logistic regression for the normalized data set, with a specificity of 0.7083 and a ROC curve of 0.8407, and Support Vector Machine with a lineal kernel for the non-normalized data; with a specificity of 0.6842 and a ROC curve of 0.7703. Both of these metrics are of utmost importance in evaluating the performance of computer-assisted diagnostic systems.

## 1. Introduction

The heart is one of the most important organs of the human body since it pumps the blood that is distributed to the entire organism through the circulatory system. This pumping process is due to the electrical and mechanical activity of the heart, which produces electrical and acoustic signals that offer information of the health of the heart and can be analyzed by physicians. By analyzing cardiac activity, it is possible to detect if the heart is working properly or if it has any pathology that is affecting the blood flow.

According to data from the World Health Organization, cardiovascular diseases (CVDs) are the leading cause of death worldwide. Annually more people die from CVDs than from any other cause, accounting 17.9 million deaths in 2016, which represent the 26.7% of the total mortality rate [1]. In Mexico, the National Institute of Geography and Statistics also reported that the main cause of death in the country was heart diseases in 2018, 149,368 deaths were registered, which represent a prevalence of the 20.6% of the mortality rate of the country [2]. For the next few years, it is estimated that CVDs will continue to be leading causes of death nationally and globally. The cardiovascular diseases can be occasioned by risk factors such as unhealthy diet and obesity, diabetes, sedentary habits, smoking and alcoholism [3]. These factors and the CVDs can be prevented and detected, an early detection and management using counseling and medicines, as appropriate, could save many lives.

Several medical devices and methods are used to detect and diagnose the CVDs both individually and together, but due to its qualities, properties and its low cost of implementation the stethoscope is still the first screening tool utilized by primary care providers for auscultatory examination [4,5]. It is an inexpensive, widely available tool in the detection of heart disease. The use of the stethoscope requires an adequate technique to hear the cardiac cycle, extensive knowledge of normal sounds and an auditory training to identify the presence of heart diseases [6]. It is easy for an experienced physician to interpret normal and abnormal sounds, but most primary care providers are unable to identify and interpret heart sounds accurately, leading to missed diagnosis of CVDs on first contact with the patient [7,8,9].

The electronic stethoscope was created to improve cardiac auscultation; it implements digital signal processing and audio enhancement techniques so that the physician is capable to hear heart sounds more clearly. Some of its advantages are that it amplifies heart sound 24–30 times, eliminates up to 85% of ambient noise, reduces friction noise between device and patient on auscultation, stores acquired audios and displays a cyclostationary signal called phonocardiogram (PCG) that represents the recorded sounds [10,11]. Using electronic stethoscopes requires to have the right technique, knowledge, and a trained ear to identify and interpret heart sounds. Furthermore, both classic and electric stethoscope are operator dependent, that is, the user must have knowledge and experience in the area to make a diagnosis [12]. In some cases, many patients with heart disease are not detected promptly [13,14,15], because primary care providers are not trained to diagnose them. In other cases, these diseases are detected at very advanced stages [16,17], in which even with the proper treatment it is difficult to reverse the damage caused by various diseases [18].

In addition to the problems presented by auscultation devices, the human auditory system is not capable to perceive all the sounds emitted by the heart; it only detects a small part of the acoustic energy generated by cardiac activity [19]. Only a small part of the sound pressure levels produced by heart sounds and murmurs in different frequency ranges can be heard, which are above the audible limit of the human auditory system [20].

Given the limitations of the human auditory system and the improper techniques in the use of the stethoscope, there is a high probability of misinterpreting heart sounds and giving erroneous diagnoses. The Institute of Medicine defines a diagnostic error as the failure to establish an accurate and timely explanation of the patient’s health problem(s) [21], while Schiff and colleagues define it as any mistake or failure in the diagnostic process leading to a wrong diagnosis, which occurs when one disease is diagnosed instead of another because they have similar signs and symptoms; a missed diagnosis, which refers to a patient whose medical complaints are never explained, as well as patients with more specific complaints that are never accurately diagnosed; or a delayed diagnosis, a case where the diagnosis is not on time, causing the disease to worsen [22].

In order to avoid diagnostic errors on cardiac auscultation, it is necessary to develop low-cost automatic diagnostic systems, known as computer-aided diagnostic (CADx) [23,24]. Its use has increased in recent years, due to the support they provide to physicians and other health care professionals in the interpretation and diagnosis of tests that the patient has undergone. The CADx have the potential to become a cost-effective screening and diagnostic tool in the primary care setting. However, it is pertinent to continue investigating and developing these systems to reduce human factor error in diagnosis of heart disease.

Computed-aided heart auscultation (CAA) is a system of automated heart sound analysis, which allows to record, visualize, store and analyze phonocardiograms [19,25,26,27,28,29]. It is also known as computerized assisted auscultation, and it has several advantages over the auscultation performed by physicians with a classic stethoscope: It helps doctors to make a more accurate and objective diagnosis of the patient’s heart health, since it is likely to outperform the auscultation skills and subjective interpretation of humans [30]; it facilitates cardiac auscultation, since not only doctors are capable of performing it, but also other health care providers can inspect correctly the patients; it has an important use in telemedicine since a physician that is somewhere in the world can diagnose the patient’s heart health in real time who is somewhere else [27,31]; the analysis results can be stored in a electronic patient record, which can be retrieved for subsequent patient appointments or for teaching and training purposes with medical students [32,33].

Computer assisted heart auscultation systems have different methods to analyze and classify heart sounds; it depends on the setting that the researcher believes is most convenient for the performance of the system and which provides the best results according to the intended purpose. However, there are steps that could be fundamental for a computer assisted heart auscultation system: pre-processing, which involves the filtering and enhancement of the cardiac sound signal, noise reduction; feature extraction, to characterize the signals; modeling, for signal reconstruction; classification, to predict whether the analyzed heart sound is normal or abnormal; and evaluation, where the performance of the classification model is measured.

The present work focuses on computer-assisted diagnosis to determine the presence or absence of heart diseases. Six Machine Learning classification methods with different metaparameters were implemented, evaluated and compared bought each other, to determine which of them better diagnoses heart audio signals as normal or abnormal sounds according to the results obtained in various evaluation metrics. The methods implemented were k-Nearest Neighbors (k-NN), Naive Bayes (NB), Decision Trees (DT), Logistic Regression (LR), Support Vector Machine (SVM) and Artificial Neural Networks (ANN).

The structure of this paper is divided into Introduction in Section 1, Materials and Methods in Section 2, Results in Section 3, Discussion in Section 4 and Conclusions in Section 5.

## 2. Materials and Methods

**Figure 1:**
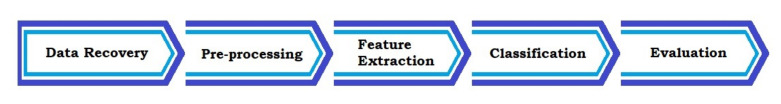
Flowchart of the methodology proposed.

In this section are described in detail dataset, features, classification methods and evaluation metrics. The methodology that was carried out in this work is represented by the flowchart showed in the Figure 1. At first, the data is recovered from the Classifying Heart Sounds Challenge. The data was pre-processed for the extraction of temporal and frequency features, which were used to classify the acoustic signals of the heart with different machine learning classification methods. Finally, the classifiers were evaluated with several metrics to analyze them from different perspectives.

### 2.1. Database Acquisition

Classifying Heart Sounds Challenge public database [34] was used to classify normal and abnormal heart sound, and it is available in http://www.peterjbentley.com/heartchallenge/#taskoverview, accessed on 25 July 2018. This database contains a total of 312 audio heart files, which were recorded with a digital stethoscope DigiScope and gathered from different individuals who underwent clinical trials in hospitals. The audio files were saved in “.wav” format and divided in three different categories depending on the state of health or heart disease; 200 files are labeled as normal heart sounds, 66 as heart murmur sounds and the remaining 46 as extra-systolic sounds.

### 2.2. Database Pre-Processing

In order to have a classification of the data as healthy or unhealthy cases, the normal heart sounds were considered as healthy heart cases, and the murmur and extra-systolic sounds as unhealthy heart cases. Since the number of healthy cases (200) almost double the unhealthy cases (112), 88 audios of normal heart sounds were randomly deleted to balance the dataset. Only 112 audios of the 200 audios of healthy sounds were considered, providing an equal number of cases of healthy and unhealthy heart sounds for analysis. In addition, because all audio files have different duration, a sub-sampling of the total audio data was performed. For practical purposes in feature extraction, the audios whose duration exceeded four seconds were selected and the rest of the audios were discarded. After this sub-sampling, the number of audios per category was as follows: 83 normal heart sounds, 47 heart murmur sounds and 31 extra-systolic sounds, that is, 83 healthy cases and 78 unhealthy cases (161 observations in total).

### 2.3. Feature Extraction

Eighteen statistical features were extracted for each of the 161 audio samples in time domain, such as mean, median, standard deviation, variance, coefficient of variation, inverse coefficient of variation, kurtosis, skewness, min value, max value, dynamic range, 1st percentile, 5th percentile, 95th percentile, 99th percentile, 1st quartile, 3th quartile and interquartile range. The same statistical features were extracted from each audio signal in frequency domain, to get a total of 36 statistical features per observation. In addition, 8 MFCC and 8 LPC coefficients were extracted.

**Figure 2:**
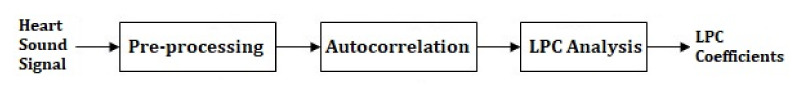
Block diagram for the calculation of the Linear Predictive Coding (LPC) coefficients.

Linear Predictive Coding (LPC) is based on the fact that each audio sample can be predicted or represented by a linear combination of several samples passed, that is, that each audio sample s(n) at a time *n*, can be approximated as a linear combination of the previous audio samples:

(1) $$s\left(n\right)\approx a_{1}s\left(n-1\right)+a_{2}s\left(n-2\right)+\cdots +a_{p}s\left(n-p\right),$$

where *p* is the prediction order and $a_{1},a_{2},\cdots ,a_{p}$ are the prediction coefficients that must be calculated. The basic diagram for calculating LPC is composed of three blocks according to Ferue et al. [35] pre-processing, autocorrelation and LPC analysis, as shown in Figure 2.

In this research, no pre-processing stage was performed in order not to modify the audio signals acquired by the stethoscope. Each of the 161 audios were autocorrelated to analyze the periodicity of the samples that comprise them. Once autocorrelated, each audio was converted into a set of LPC coefficients by the Levinson–Durbin autoregressive algorithm. Eight LPC coefficients were calculated per audio sample, which represent the information of the short-time spectral envelope of the audio signals according to Wang et al. [36].

**Figure 3:**
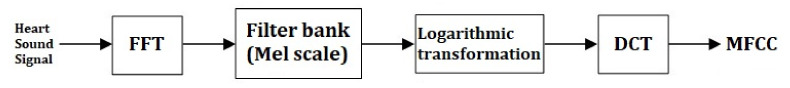
Block diagram for the calculation of the Cepstral Frequency–Mel Coefficients (MFCC).

Cepstral Frequency–Mel Coefficients (MFCC) is a feature extraction technique that is based on the perception of the human auditory system, specifically the variation of the bandwidths of the critical frequencies of the human ear. The basic method for the extraction of the MFCC is composed of four blocks according to Mascorro et al. [37]: Fast Fourier Transform (FFT), filter bank, logarithm transformation and Discrete Cosine Transform (DCT), as shown in Figure 3.

In this extraction technique, as in LPC, no pre-processing stage was performed either. In the first block, the FFT of each of the cardiac audio signal was calculated, and its magnitude and power spectral density (PED) were obtained. This transformation was performed to identify which frequencies each of the audio signals contains. The DSP frequencies must be grouped into regions and added together to find out how much energy exists in those regions. This was done by means of a Mel filter bank, made up of triangular filters that are distributed in Mel scale. The filter bank was calculated with the Equation (2).

(2) $$B\left(m,k\right)=\left\{\begin{array}{ccc} 0 & si & k>f(m-1),\\ \frac{k-f(m-1)}{f(m)-f(m-1)} & si & f(m-1)\leq k\leq f(m),\\ \frac{f(m+1)-k}{f(m+1)-f(m)} & si & f(m)\leq k\leq f(m+1),\\ 0 & si & k>f(m+1), \end{array}\right.$$

where B(m,k) is the matrix of the filter bank, *m* the number of bank filters and *k* the number of analysis windows (one in this case). A filter bank was obtained for each audio signal. The first filter obtained is very narrow and indicates how much energy exists near 0 Hz. As the frequencies increase, the filters expand and the variations are smaller. To know the energy of the filter banks, we must multiply each bank of filters by the power spectral density windows and then add the coefficients:

(3) $$E\left(m,k\right)=\sum_{m=1}^{M}B\left(m,k\right)P\left(k\right)\;k=1,$$

where P(k) is the power spectral density and *k* represents the number of windows again. Subsequently, the logarithm of the filter bank energies was calculated. This operation makes the obtained characteristics a closer match to what humans actually hear.

(4) $$E_{log}\left(m,k\right)=log\left(\sum_{m=1}^{M}B\left(m,k\right)P\left(k\right)\right)$$

In the last block, the discrete cosine transform of the logarithm of the filter bank energies was calculated to obtain the MFCC [38]. The use of the DCT is used to reduce computational complexity [39], and it is defined by

(5) $$MFCC\left(n\right)=\sum_{m=1}^{M}E_{log}\left(m,k\right)cos\left[n\left(m-\frac{1}{2}\right)\frac{\pi }{M}\right],$$

where *M* represents the total number of MFCC coefficients that varies with respect to *n*, *m* represents the number of filters in the bank, and *k* represents the number of the analysis window. As in LPC, 8 MFCC coefficients were calculated per audio signal.

Once the 36 statistical features, the 8 LPC coefficients and the 8 MFCC coefficients for each of the cardiac audio signals were calculated, they were used to form a dataset of 52 features and 161 observations. From this dataset, two more were obtained, one normalized and one standardized. In the normalized dataset, the values of each feature are in a range from zero to one and were obtained according to the Equation (6).

(6) $$X_{norm}=\frac{X-min(X)}{max(X)-min(X)}$$

where $X_{norm}$ is the normalized value, and *X* is the value without normalization. The standardized dataset was obtained with/ the z-score method, in which the values of each feature do not have a defined minimum or maximum value but a mean always equal to zero. The z-score standardization is obtained with the Equation (7)

(7) $$X_{stand}=\frac{X-\mu }{\sigma }$$

where $X_{stand}$ is the standardized value, and *X* is the value without standardization, μ and σ are the mean and the standard deviation of the value without standardization, respectively. These three datasets were used to classify heart sounds, using 75% of the data for training and 25% for testing.

### 2.4. Classification Methods

Six different classification methods were implemented in the programming language R to classify cardiac sounds as healthy or unhealthy:

- k-Nearest Neighbors: This classifies unlabeled examples according to known classified neighbors. The letter *k* represents a variable term that specifies the number of closest neighbors. This number starts with a value of *k* equal to an odd number approximately equal to the square root of the number of training examples; the odd number is used to eliminate the possibility of ending with a tie [40,41,42,43]. For the implementation of this classification method, the *class* library and the *knn* function were used. Seven different k-neighbors were used: 1, 5, 11, 13, 15, 21 and 27.
- Naive Bayes: This uses training data to calculate an observed probability of each outcome based on the evidence provided by the values of the characteristics When the classifier is later applied to unlabeled data, it uses the observed probabilities to predict the most probable class for the new characteristics. Naive Bayes model was implemented using the *e1072* library and the *naiveBayes* function. Two configurations were made for this classifier, one with a Laplacian estimator and the other without it. This estimator adds a small number to each of the values in the frequency table to guarantee that each characteristic has a probability other than zero for any of the classes.
- Decision Trees: This is a powerful classifier that uses a tree structure to model the relationship between features and potential outcomes. A decision tree classifier uses a branching decision structure, which pipes examples to predict a final class value. The *c50* library was used to implement the decision trees model. Three different decision tree configurations were set: one with indefinitely growing of the branches, one with a post-pruning of the branches to reduce the size of the tree and another with a cost error in the confusion matrix in order to avoid false negatives.
- Logistic Regression: It studies the relationship between a categorical dependent variable and a set of independent variables. It is so named because the dependent variable has only two possible values, 0 and 1 or “yes” and “no”. This technique uses the one versus the rest (OvR) scheme to predict the probability of a categorical dependent variable [44,45,46]. A function of generalized linear models *glm* with a logistic regression setup was implemented.
- Support Vector Machine: This can be thought of as a surface that defines a boundary between several data points representing examples drawn in multidimensional space according to their characteristic values. The goal of an SVM is to create a flat boundary, called a hyperplane, which leads to fairly homogeneous data partitions on both sides [47,48,49,50,51]. To implement the Support Vector Machine model, the *kernlab* library and the *ksvm* function were applied, and four different seed kernels were used to compare different perspectives of the data distribution: linear, radial basis, polynomial and hyperbolic tangent sigmoid.
- Artificial Neural Networks: This is an information processing method based on the system that the brain involves to process information. It models the interconnections of neurons in a brain using artificial neurons known as nodes, which relate an input signal and an output signal. Each node contains an activation function that has the function of thresholding the values of the nodes to take them to any of the possible results [52,53,54,55,56]. The *neuralnet* library was used to implement the Artificial Neural Networks model. Three different ANN architectures were configured: a ANN with one hidden layer and one neuron, a ANN with one hidden layer and seven neurons, and a ANN with two hidden layers and twelve neurons in the firs layer and four in the second. The three topologies had the same number of input neurons (fifty two) and output neurons (two). The three ANNs used the same linear activation function.

### 2.5. Evaluation Metrics

For the evaluation of the six classification models with their different configurations, the *gmodel*, *pROC* and *caret* libraries were used, with which it was possible to calculate the following metrics:

- Accuracy is the percentage of classifying positive and negative samples correctly [57,58]. It is calculated as shown in the Equation (8).
  (8) $$Accuracy=\frac{TruePositives+TrueNegatives}{TotalExamples}$$
- Sensitivity (true positive rate) measures the proportion of true positives that are correctly identified as such, that is, of all patients who are sick, how many are correctly detected as sick [59,60]. It is calculated as shown in the Equation (9).
  (9) $$Sensitivity=\frac{TruePositives}{FalseNegative+TruePositives}$$
- Specificity (rate of true negatives) measures the proportion of real negatives that are correctly identified as such, that is, of all patients who are not sick, how many were correctly detected as not ill [61]. It is calculated as shown in the Equation (10).
  (10) $$Specificity=\frac{TrueNegatives}{FalsePositive+TrueNegative}$$
- Precision (positive predictive value) measures the consistency of results when measurements are repeated [62,63,64]. It is calculated as shown in the Equation (11).
  (11) $$Precision=\frac{TruePositives}{FalsePositive+TruePositives}$$
- F1 score is the harmonic mean of the precision and sensitivity [65,66]. It is calculated as shown in the Equation (12).
  (12) $$F1S=2\frac{Precision*Sensitivity}{Precision+Sensitivity}$$
- ROC curve (Receiver Operating Characteristics) provides a global measure of diagnostic precision, independent of the cut-off point and prevalence. It is obtained by representing the sensitivity (percentage of true positives) on the ordinate axis and 1-Specificity (percentage of false positives) on the abscissa axis, for different cut-off points applied to the quantitative result of a test [67,68].

## 3. Results

The obtained results by each classifier in the different evaluation metrics are presented in three different tables: Table 1 shows the results of the dataset with the values of the extracted features (for convenience it will be called original dataset); Table 2 shows the results of the data set with the normalized values, and Table 3 shows the results of the data set with the standardized values. The three tables contain the same number of columns, the first one correspond to the name of the classification method, the next six to the evaluation metrics; accuracy, specificity, sensitivity, area under the curve (ROC), precision and F1-score, respectively, and the last one to the mean of the evaluation metrics (EM Mean) for each classifier. The tables also have the same number of rows, which represent each classification method: k-nearest neighbor (k-NN) with seven different number of neighbors (k); Naive Bayes with laplacian (NB with Δ) and without it (NB w/o Δ); Decision Trees without pruning (DT w/o P), with pruning (DT with P), and with a cost in the confusion matrix (DT with C); Logistic Regression (LR); Support Vector machine with four different kernels, linear (SVM linear), radial (SVM radial), polynomial (SVM polynomial) and hyperbolic tangent (SVM HT); and Artificial Neural Networks, with one hidden layer and one neuron (ANN 1HL 1N), with one hidden layer and seven neurons (ANN 1HL 7N), and with two hidden layers and 12 neurons in the first layer and 4 in the second (ANN 2HL 12N 4N).

Based on literature [69,70,71,72,73,74,75,76] in the biomedical area, a comparison was made between the different models implemented according to evaluation metrics described in this area of knowledge, taking into consideration that the values of these metrics are considered better as closer they are to unit.

According to the results of the evaluation metrics obtained by the classifiers in the original dataset shown in the Table 1, the highest accuracy value 0.7073 was presented in three classifiers; SVM with a polynomial kernel, SVM with a linear kernel and K-NN with *k* = 27. Furthermore, SVM with a linear kernel had the highest values of ROC 0.7703 and EM Mean 0.7293, and K-NN with *k* = 27 got the highest value of F1-score 0.7692. Naive Bayes classifier with and without Laplacian presented the highest values of specificity and precision, 0.8667 and 0.8333, respectively. The highest value of sensitivity 1 was obtained by ANN 1HL 1N, but it also got the worst specificity, ROC and precision values. The lowest accuracy was obtained by K-NN with *k* = 5 and ANN 1HL 7N, in addition, K-NN with *k* = 5 obtained the lowest EM Mean and ANN 1HL 7N the lowest values of sensitivity and F1 score.

Analyzing the results of the evaluation metrics of each classifier in the normalized dataset (shown in Table 2), the classifier with the highest accuracy value 0.7317 was Logistic Regression, which also obtained the highest ROC 0.8407. Decision trees without pruning was the method with the best specificity 0.8261, but it obtained the lowest values in terms of sensitivity and F1 score. The classifier with the best sensitivity 0.92 was K-NN with *k* = 27, but got the lowest precision and a poorly specificity. ANN 1HL 1N obtained the highest values of precision 0.75 and F1-score 0.7777. ANN 1HL 7N got the lowest accuracy value. DT with C got the lowest specificity and ROC values. The lowest EM Mean was obtained by K-NN with *k* = 13 and *k* = 15.

The results of the evaluation metrics obtained by the classifiers in the standardized dataset (shown in Table 3) were similar to ones obtained in the normalized dataset. It was found that the Logistic Regression model had the highest values in EM Mean and three metrics: accuracy 0.8049, ROC 0.8405 and F1-score 0.8181. For specificity, the highest value was 0.8261 obtained by the model DT w/o pruning, and DT with C had the best sensitivity 0.8846. ANN 2HL 12N 4N got the best precision value 0.85, and this method obtained values above 0.7 in all metrics, with the exception of 0.6538 in sensitivity. SVM HT got the lowest values of accuracy, specificity, precision, F1- score and obviously EM Mean. The k-NN with *k* = 1 had the lowest sensitivity and ROC values.

## 4. Discussion

According to the results shown in Table 1, the best classifier for the original dataset was SVM with a linear kernel. It had the highest accuracy, ROC and EV mean values, and the other values obtained were above the mean of all classifiers. For the normalized dataset, the best classifier was the Logical Regression model. This method also got the maximum values of accuracy, ROC and EV mean as SVM with a linear kernel in the original dataset. A very similar case happened with the standardized dataset; the best classifier was Logistic Regression. In addition to obtaining the maximum values in the same evaluation metrics as in the previous datasets, the maximum value of F1-score was also obtained by this method.

Accuracy and ROC are important metrics in computer-assisted diagnosis, since the first represents the percentage of normal and abnormal heart sounds classified correctly, and the second represents globally the precision of the diagnosis, that is, how many healthy sounds were diagnosed as such and how many not healthy were diagnosed as such.

Another very important metric for CADx, which is directly related to the ROC curve is specificity, since it indicates the proportion of actual healthy sounds that are correctly identified as such. This metric is important, since it is better to tell a healthy person that is sick than to tell a sick person that is healthy. Since this would complicate the illness or cause death. However, the classifiers that had the maximum values of specificity had in turn a very insignificant value of sensitivity Naive Bayes with and without Laplacian for the original dataset and Decision Trees without pruning for the normalized and standardized datasets. This means that there may be abnormal heart sounds classified as normal.

Comparing the results obtained by the best classifier of each of the three datasets, it was found that Logical Regression model in the standardized dataset performs better in most of the evaluation metrics values respect to Logical Regression model in the normalized dataset and Support Vector Machines with a linear kernel in the original dataset. Only the ROC value was identical for the Logistic Regression in the normalized and standardized datasets. Furthermore, it was found that the ANN 2HL 12N 4N improved considerably its evaluation metrics values in the standardized dataset compared to the other two datasets. It is important to highlight that although the logistic regression algorithm is the simplest of the implemented algorithms, it presented the best results according to the evaluation metrics. This may be due to its nature of finding a positive and a negative class.

Using the same audio database Ferreira and Pereira [77] got a 0.4566 precision using an algorithm based on decision trees (J48), while using an Artificial Neural Network with multiple layers (MLB-Multi Layer Perceptron) got a 0.5566 precision. Deng and Bentley [78] also used the same audio database and classified the audio files according to their number of beats per minute (N. BPM) by setting a heartbeat threshold for each heart sound class; they got a 0.4377 precision. However, these two works made a classification of three classes: normal, extrasystolic and murmur sounds, unlike our classification that was of two classes: normal and abnormal heart sounds. For a comparison from the same approach of the three works, the results obtained in the precision evaluation metric are shown in Table 4.

Table 4 shows the precision values obtained in the standardized database of three classifiers, Logistic Regression, Decision Trees without Pruning and Artificial Neural Networks with two hidden layers, which are better compared to those obtained by the J48 and MLB approaches of Ferreira and Pereira and the N. BPM approach of Deng and Bentley. The precision values presented by these two works were obtained from each of the classes: precision of normal, precision of murmur and precision of extrasystolic sounds. Therefore, the average of them was calculated to be comparable with the precision values obtained in our classification models. The LR classifier was compared since it was the one that presented the best results, and the DT w/o P and ANN 2HL 12N 4N classifiers were compared because they present similar approaches to those used by Ferreira and Pereira.

In both works with which ours is being compared, pre-processing and processing of the audio signals is carried out, and the results obtained are below those obtained by our work. The fundamental contribution of this work lies in the fact that the classification of cardiac audio signals as normal or abnormal is done without the signal acquired by the stethoscope being modified in the pre-processing or processing stages; only temporal and frequency characteristics are extracted from the nature of the cardiac acoustic signal. This directly reduces computational expense, and in turn, allows new cardiac audio signals to be classified in the same way.

As it can be observed in Table 5, the values obtained of specificity and sensitivity in this work are better respect to the ones of the other two research. However, they cannot be compared directly since in our case, both training and test sets are balanced; that means that have approximately the same number of sick cases and controls, and all evaluation metrics were obtained from the complete test set, while the other two investigations consider only the subgroup of patients with heart disease to calculate the specificity and sensitivity. This allows to generalize that our work simulates a scenario close to reality, where it is unknown whether the patient to be analyzed is sick or not, in order to avoid biases and overfitting problems.

## 5. Conclusions

Despite the fact that various methods have emerged to try to classify heart diseases [25], this study has advantages such as that the sounds used have the characteristics that real-time models would face, since it was avoided to use pre-processing, to generate an environment closer to reality. It allows to accelerate the analysis process and the possibility of using this type of classification method in devices not so complex to use, in order to make them affordable for health personnel, even in marginalized areas, for easy, accurate and timely identification of heart diseases, where it is unlikely that there will be specialists in these pathologies, and at the same time, it will contribute to carrying out the timely referrals to the second and third levels of health, as appropriate, avoiding unnecessary shipments or the absence of timely referrals to the specialist [30], on the other hand, expedites the consultation of first contact, reducing workloads on health professionals.

After having tested the classification methods, to determine if a patient is healthy or sick, it is clear that the results obtained (see Table 1, Table 2 and Table 3) are statistically significant, which allows the experiments carried out in this work to be reproducible. By following the same steps and using the same data, the same results can be obtained. However, as mentioned in the literature, there is controversy in the term reproducible and replicable [79], but taking one or the other, what stands out is that the experiments can obtain the same results, following the same steps with different data (heart sounds). That is, with the methodology proposed in this work, it is intended to be scalable so that in the future, a computer-assisted diagnosis may be available, in which health personnel introduce sounds as an input source, and the system provides a true diagnosis.

Some of the limitations that can be faced when implementing these classification methods in real life are the following: Specialized equipment is required such as electronic stethoscopes, as well as a computers with the R software and the required packages (described in materials and methods) installed to implement each of the classifiers and diagnose the patient as healthy or sick. Another limitation is the data with which they were worked, which could present population biases, so the models would benefit from having a more robust training database that includes patients of all ages and physiological conditions such as pregnancy and old age, among others. In the future, tools that allow the physician–machine interaction can be designed to improve user usability, and this allows it to be used in real time as a support for computer-assisted diagnosis.

In order to improve the results obtained in this work, it is necessary to add a feature selection stage using genetic algorithms. This would allow us to consider the most significant features and improve the performance of the classifiers. In addition, using deep learning classification models would also allow us to improve the classification of heart sounds; however, it would increase computational expenditure and would be reflected in real-time diagnosis. 

## Figures and Tables

**Table 1:** Evaluation metrics for each classifier of the original dataset.

Classification Method	Accuracy	Specificity	Sensitivity	ROC	Precision	F1-Score	EM Mean
k-NN, k=1	0.6585	0.6667	0.6522	0.6594	0.7143	0.6818	0.6721
k-NN, k=5	0.561	0.5556	0.5652	0.5604	0.6190	0.5909	0.5753
k-NN, k=11	0.6585	0.6111	0.6957	0.6534	0.6957	0.6956	0.6683
k-NN, k=13	0.6098	0.5556	0.6522	0.6039	0.6522	0.6521	0.6209
k-NN, k=15	0.6098	0.5556	0.6522	0.6039	0.6522	0.6521	0.6209
k-NN, k=21	0.6098	0.5000	0.6957	0.5978	0.6400	0.6666	0.6183
k-NN, k=27	**0.7073**	0.5000	0.8696	0.5978	0.6897	**0.7692**	0.6889
NB w/o Δ	0.5897	**0.8667**	0.4167	0.6417	**0.8333**	0.5555	0.6506
NB with Δ	0.5897	**0.8667**	0.4167	0.6417	**0.8333**	0.5555	0.6506
DT w/o P	0.6383	0.8261	0.4583	0.6422	0.7333	0.5641	0.6437
DT with P	0.617	0.6364	0.6000	0.6182	0.6522	0.625	0.6248
DT with C	0.6383	0.3333	0.8846	0.609	0.6216	0.7301	0.6361
LR	0.6327	0.7222	0.5806	0.7204	0.7826	0.6666	0.6841
SVM linear	**0.7073**	0.6842	0.7273	**0.7703**	0.7273	0.7272	**0.7293**
SVM radial	0.6829	0.5789	0.7727	0.7344	0.6800	0.7234	0.6953
SVM polynomial	**0.7073**	0.6316	0.7727	0.7536	0.7083	0.7111	0.7141
SVM HT	0.6341	0.6316	0.6364	0.6053	0.6667	0.6511	0.6375
ANN 1HL 1N	0.5854	0.1053	**1.0000**	0.5526	0.5641	0.7213	0.5881
ANN 1HL 7N	0.561	0.8421	0.3182	0.6352	0.7000	0.4375	0.5881
ANN 2HL 12N 4N	0.6829	0.7895	0.5909	0.6902	0.7647	0.6666	0.6974

Abbreviations: ROC: Receiver Operating Characteristics curve, EM: evaluation metrics, k-NN: k-nearest neighbor, *k*: number of neighbors, NB: Naive Bayes, Δ:laplacian, w/o: without, DT: Decision Trees, P: pruning, C: cost in the confusion matrix, LR: Logistic Regression, SVM: Support Vector Machine with linear, radial, polynomial and hyperbolic tangent (HT) kernels, ANN: Artificial Neural Networks, HL: hidden layers and N: number of neurons per hidden layer. Numbers in bold represent the maximum values obtained for each evaluation metric by the different classifiers.

**Table 2:** Evaluation metrics for each classifier of the normalized dataset.

Classification Method	Accuracy	Specificity	Sensitivity	ROC	Precision	F1-Score	EM Mean
K-NN, k=1	0.6531	0.5833	0.7200	0.6517	0.6429	0.6792	0.6550
K-NN, k=5	0.6327	0.5417	0.7200	0.6308	0.6207	0.6666	0.6354
K-NN, k=11	0.6327	0.5000	0.7600	0.63	0.6129	0.6785	0.6356
K-NN, k=13	0.6122	0.5417	0.6800	0.6108	0.6071	0.6415	0.6155
K-NN, k=15	0.6122	0.5417	0.6800	0.6108	0.6071	0.6415	0.6155
K-NN, k=21	0.6531	0.4167	0.8800	0.6483	0.6111	0.7213	0.6550
K-NN, k=27	0.6531	0.3750	**0.9200**	0.6475	0.6053	0.7301	0.6551
NB w/o Δ	0.6923	0.8000	0.5789	0.6895	0.7333	0.6470	0.6901
NB with Δ	0.6923	0.8000	0.5789	0.6895	0.7333	0.6470	0.6901
DT w/o P	0.6383	**0.8261**	0.4583	0.6422	0.7333	0.5641	0.6437
DT with P	0.617	0.6364	0.6000	0.6182	0.6522	0.625	0.6248
DT with C	0.6383	0.3333	0.8846	0.609	0.6216	0.7301	0.6361
LR	**0.7317**	0.7083	0.7647	**0.8407**	0.6500	0.7027	**0.7330**
SVM linear	0.6585	0.4211	0.8636	0.7512	0.6522	0.7307	0.6795
SVM radial	0.7073	0.4737	0.9091	0.7871	0.6667	0.7692	0.7188
SVM polynomial	0.6829	0.4737	0.8636	0.7512	0.6552	0.7450	0.6952
SVM HT	0.6098	0.7368	0.5000	0.6364	0.6875	0.5789	0.6249
ANN 1HL 1N	0.7073	0.5333	0.8077	0.6308	**0.7500**	**0.7777**	0.7011
ANN 1HL 7N	0.5854	0.6667	0.5385	0.6308	0.7368	0.6222	0.6300
ANN 2HL 12N 4N	0.6098	0.5333	0.6538	0.6821	0.7083	0.68	0.6445

Abbreviations: ROC: Receiver Operating Characteristics curve, EM: evaluation metrics, k-NN: k-nearest neighbor, *k*: number of neighbors, NB: Naive Bayes, Δ:laplacian, w/o: without, DT: Decision Trees, P: pruning, C: cost in the confusion matrix, LR: Logistic Regression, SVM: Support Vector Machine with linear, radial, polynomial and hyperbolic tangent (HT) kernels, ANN: Artificial Neural Networks, HL: hidden layers and N: number of neurons per hidden layer. Numbers in bold represent the maximum values obtained for each evaluation metric by the different classifiers.

**Table 3:** Evaluation metrics for each classifier of the standardized dataset.

Classification Method	Accuracy	Specificity	Sensitivity	ROC	Precision	F1-Score	EM Mean
K-NN, k=1	0.4694	0.5600	0.3750	0.5276	0.4828	0.5660	0.4968
K-NN, k=5	0.6341	0.6250	0.6400	0.6325	0.7273	0.6808	0.6566
K-NN, k=11	0.6341	0.6250	0.6400	0.6325	0.7273	0.6808	0.6566
K-NN, k=13	0.6341	0.6250	0.6400	0.6325	0.7273	0.6808	0.6566
K-NN, k=15	0.6531	0.5217	0.7692	0.6455	0.6452	0.7017	0.6560
K-NN, k=21	0.6098	0.5000	0.6800	0.5900	0.6800	0.6800	0.6233
K-NN, k=27	0.6341	0.5625	0.6800	0.6212	0.7083	0.6938	0.6499
NB w/o Δ	0.6667	0.7727	0.5294	0.6511	0.6429	0.5806	0.6405
NB with Δ	0.6667	0.7727	0.5294	0.6511	0.6429	0.5806	0.6405
DT w/o P	0.6383	**0.8261**	0.4583	0.6422	0.7333	0.5641	0.6437
DT with P	0.617	0.6364	0.6000	0.6182	0.6522	0.6250	0.6248
DT with C	0.6383	0.3333	**0.8846**	0.609	0.6216	0.7301	0.6361
LR	**0.8049**	0.7500	0.8571	**0.8405**	0.7826	**0.8181**	**0.8088**
SVM linear	0.7073	0.6364	0.7895	0.6962	0.6522	0.7142	0.6993
SVM radial	0.6585	0.5455	0.7895	0.6986	0.6000	0.6818	0.6623
SVM polynomial	0.7073	0.6364	0.7895	0.6962	0.6522	0.7142	0.6993
SVM HT	0.4146	0.2273	0.6316	0.5646	0.4138	0.5000	0.4586
ANN 1HL 1N	0.6341	0.7778	0.5217	0.7029	0.7500	0.6153	0.6669
ANN 1HL 7N	0.6341	0.6667	0.6154	0.6308	0.7619	0.6808	0.6700
ANN 2HL 12N 4N	0.7073	0.8000	0.6538	0.7513	**0.8500**	0.7391	0.7502

Abbreviations: LR: Logistic Regression, DT w/o P: Decision Trees without pruning, ANN 2HL 12N 4N: Artificial Neural Networks with two hidden layers and twelve neurons in the first layer and four in the second, J48: algorithm based on decision trees, MLB: Multi Layer Perceptron, N.BPM: algorithm based on the number of beats per minute. Abbreviations: ROC: Receiver Operating Characteristics curve, EM: evaluation metrics, k-NN: k-nearest neighbor, *k*: number of neighbors, NB: Naive Bayes, Δ:laplacian, w/o: without, DT: Decision Trees, P: pruning, C: cost in the confusion matrix, LR: Logistic Regression, SVM: Support Vector Machine with linear, radial, polynomial and hyperbolic tangent (HT) kernels, ANN: Artificial Neural Networks, HL: hidden layers and N: number of neurons per hidden layer. Numbers in bold represent the maximum values obtained for each evaluation metric by the different classifiers.

**Table 4:** Comparison of our results with respect to other works that used the same audio database.

	LR	DT w/o P	ANN 2HL 12N 4N	J48	MLB	N. BPM
Precision	0.7826	0.7333	**0.8500**	0.4566	0.5566	0.4377

Abbreviations: LR: Logistic Regression, DT w/o P: Decision Trees without pruning, ANN 2HL 12N 4N: Artificial Neural Networks with two hidden layers and twelve neurons in the first layer and four in the second, J48: algorithm based on decision trees, MLB: Multi Layer Perceptron, N.BPM: algorithm based on the number of beats per minute. Number in bold represents the maximum values obtained for each evaluation metric by the different classifiers.

**Table 5:** Sensibility and Specificity values.

	LR	DT w/o P	ANN 2HL 12N 4N	J48	MLB	N. BPM
Sensitivity	0.8571	0.4583	0.6538	0.22	0.19	0.5085
Specificity	0.75	0.8261	0.8000	0.82	0.84	0.5882

Abbreviations: LR: Logistic Regression, DT w/o P: Decision Trees without pruning, ANN 2HL 12N 4N: Artificial Neural Networks with two hidden layers and twelve neurons in the first layer and four in the second, J48: algorithm based on decision trees, MLB: Multi Layer Perceptron, N.BPM: algorithm based on the number of beats per minute.

## Data Availability

Publicly available datasets were analyzed in this study. This data can be found here: http://www.peterjbentley.com/heartchallenge/#taskoverview.
